# Suilyin Disrupts the Blood–Brain Barrier by Activating Group III Secretory Phospholipase A2

**DOI:** 10.3390/life12060919

**Published:** 2022-06-20

**Authors:** Yutong Sui, Ying Chen, Qingyu Lv, Yuling Zheng, Decong Kong, Hua Jiang, Wenhua Huang, Yuhao Ren, Peng Liu, Yongqiang Jiang

**Affiliations:** 1 State Key Laboratory of Pathogen and Biosecurity, Beijing Institute of Microbiology and Epidemiology, Academy of Military Medical Sciences (AMMS), Beijing 100071, China; suiyt888@163.com (Y.S.); lvqingyu2004@126.com (Q.L.); zhengyuling@sina.com (Y.Z.); kongdecong-118@163.com (D.K.); jhua76@126.com (H.J.); huangwh1993@163.com (W.H.); www2354@126.com (Y.R.); 2 School of Light Industry, Beijing Technology and Business University (BTBU), Beijing 100048, China; chenying@btbu.edu.cn

**Keywords:** *S. suis* 2, Suilysin, PLA2G3, meningitis, blood–brain barrier

## Abstract

Serious diseases caused by *Streptococcus suis* serotype 2 (*S. suis* 2) include septicaemia and meningitis, which are associated with high morbidity and mortality. Proliferation in the blood can result in a breach of the blood–brain barrier (BBB) and provide entry into the cerebrospinal fluid (CSF), where bacteria cause inflammation of the meningeal membranes resulting in meningitis. The molecular mechanisms of how this pathogen crosses the BBB remain unclear. Suilysin (SLY) has been identified as an important secreted virulence factor of *S. suis* 2 and may play a vital role in provoking meningitis. In this investigation, we demonstrate that SLY can increase the paracellular permeability of BBB, both in vivo and in vitro, via the activation of group III secretory phospholipase A2 (PLA2G3). Our results indicate that at lower, sublytic concentrations, the toxin can stimulate cerebral microvascular endothelial cells to release TNF-α, thereby inducing high level expressions of PLA2G3. Abnormal elevations of PLA2G3 might further injure tissues through direct cytolytic effectors or other responses.

## 1. Introduction

As an important emerging zoonotic pathogen that has gained more attention in recent years, *Streptococcus suis* can infect humans through close contact with pigs or fresh pork. The main clinical conditions caused by *S. suis* infection in humans are sepsis and meningitis, although other symptoms have also been described, such as endocarditis, arthritis, pneumonia, and bacteremia [1]. *S. suis* is distributed worldwide and infections by this pathogen tend to occur sporadically [2]. For instance, in 2005, an outbreak of human *S. suis* 2 infections was reported in Sichuan, China [3], and over 200 infected humans developed meningitis during the course of this epidemic [4]. Because of the high rates of resulting morbidity and mortality, bacterial meningitis is considered to be one of the ten leading cause of infection-related deaths around the world [5,6]. Similarly to other bacteria-caused cases of meningitis, the pathogenesis of *S. suis* 2 meningitis is based on complex host–pathogen interactions [7,8]. As we described previously, *S. suis* 2 can escape human innate immune responses, resulting in hematogenous spreading to the brain [9], but the mechanisms that allow circulating *S. suis* 2 to cross the blood–brain barrier (BBB) and to infect the central nervous system are still imprecisely understood [10]. Thus, in this report, we focus our attention on how *S. suis* 2 penetrates the BBB, which mostly consists of brain microvascular endothelial cells.

Several virulence factors have been identified in the pathogenesis of *S. suis* 2 infection [11]; among them, suilysin (SLY) is a well-known secreted protein. Its discovery helped confirm that SLY contributes to the development of meningitis in humans [12], but the mechanism remains unclear. As determined in our previous study, the virulent *S. suis* 2 strain 05ZYH33 induced cerebral microvascular endothelial cells (hCMEC) to release the pro-inflammatory cytokine TNF-α, while the mutant strain △SLY did not [13]. In this study, we used bacterial culture supernatant and purified native SLY (nSLY) to stimulate hCMEC and found that the induced over-expression of TNF-α was time dependent. It has been reported that TNF-α can induce the expression of secretory phospholipase A2 (sPLA2) in a variety of cells [14,15,16], but endothelial cells were not studied.

sPLA2 are members of the largest subfamily in phospholipase A2 (PLA2) superfamily, composing about 10 isozymes (group IB (G1B), IIA, IIC, IID, IIE, IIF, III, V, X and XII) described in mammals [17]. Mammalian sPLA2s have been subdivided into three structural classes based on the position of disulfide bonds and sequence alignment. Groups I, II, V, and X sPLA2 comprise one subclass and, as such, have similar primary structures and partially overlapping sets of disulfides. Mammalian GIII and GXIIA sPLA2s are atypical members of the sPLA2 family [18]. sPLA2 enzymes catalyze the hydrolysis of the *sn*-2 position of membrane glycerophospholipids, leading to the production of arachidonic acid (AA) and lysophospholipids. Lysophospholipids such as lysophosphatidic acid (LPA) and lysophosphatidylcholine (LPC) are also biologically active by themselves [19]. Furthermore, LPA has been demonstrated that it could disrupt the structural integrity of BBB [20,21].

Taking into consideration the published findings [22,23] in combination with our previous experimental results, we speculated that the subcytolytic effects of SLY on *S. suis* 2 meningitis may be associated with sPLA2 production. To test this hypothesis, we treated hCMEC monolayers with or without SLY, and changes in sPLA2 expression levels were assessed by reverse transcription quantitative PCR (RT-qPCR) and Western blot analysis. Our results indicate that hCMEC constitutively expressed low level PLA2G3, and its expression increased after a 4 h incubation with SLY. The increased expression of PLA2G3 by SLY coincided temporally with the disruption of BBB integrity and SLY-mediated PLA2G3 over-expression in hCMECs could induce apoptosis and actin cytoskeleton reconstruction; all of these abnormal cellular responses can result in gap formations between hCMECs and BBB disruption. Incubation with a secretory phospholipase A2 inhibitor or the use of PLA2G3 RNA interference blocked barrier disruption by SLY. Moreover, after the addition of the TNF-α inhibitor pomalidomide, the effect of SLY on PLA2G3 expression was significantly attenuated. These experiments confirmed that SLY does not directly induce PLA2G3 overexpression, but instead, this process is dependent on SLY induced TNF-α expression. Furthermore, we demonstrate in vivo that sPLA2 inhibition impedes *S. suis* entry into mouse brains.

## 2. Materials and Methods

### 2.1. Ethics Statement

The mice used in our study were female C57BL/6 (7 to 8 weeks-old), which were housed under SPF conditions. Our work was approved by the Institutional Animal Care and the Animal Ethics Committee of the Academy of Military Medical Sciences (AMMS). In all experiments, we tried our best to minimize the suffering of animals.

### 2.2. Strains, Cells, and Culture Conditions

The highly virulent *S. suis* 2 strain 05ZYH33 was originally isolated from a deceased patient, and the isogenic mutant statin 05ZYH33△SLY was constructed in our laboratory. The culture conditions of the stations were the same as previously described [13]. Immortalized human cerebral microvascular endothelial cells (hCMEC/D3) were generously provided by Professor Pierre-Olivier Couraud (INSERM, Paris) and cultured as described in previously published methods [24].

### 2.3. Purification of nSLY Protein and Determination of Sub-Haemolytic Concentrations

05ZYH33 and △SLY strains were cultured at 37 °C overnight in Todd–Hewitt broth (THB, Difco), and the supernatants were collected and filtered by Millipore filter (pore size, 0.22 μm). The native SLY (nSLY) protein was purified and activity was analyzed as described in our previous report [13]. By conducting optimization experiments, we decided to use 125 ng/mL suilysin in all experiments, which is clearly below the amount needed to induce cytolytic effects (Supplemental Figure S1). nSLY protein was mixed with 2% sheep red blood cells in Eppendorf tubes and incubated at 37 °C for 60 min and the supernatant absorbance of each tube was measured at OD540 using the MULTISKAN MK3 (Thermo Scientific). The amount of bacterial culture supernatant used to stimulate hCMEC/D3 is based on sub-haemolytic concentrations of nSLY protein.

### 2.4. RT-qPCR Analysis

The accumulation levels of sPLA2 or TNF-α mRNA in hCMEC/D3 cells stimulated by SLY were calculated as the average fold increase in the control group. RNA isolation and characterization was performed as previously described in detail [13]. The relative mRNA levels were determined by calculating the threshold cycle (∆ Ct) of each gene using the classic ∆Ct method. The primers used in this study are listed in Supplementary Table S1. All RNA expression levels were normalized against the expression of the reference gene GAPDH.

### 2.5. Western Blotting Analysis

HCMEC/D3 cells were stimulated with the nSLY (125 ng/mL) protein or the supernatant of *S. suis* 2 separately. After treatment, hCMECs were subsequently collected and lysed as previously described [25], and the culture supernatants were also collected. The sample proteins were separated by SDS-PAGE electrophoresis and transferred onto polyvinylidene difluoride (PVDF) membranes (Merck Millipore Corporation, Burlington, MA, USA). The membranes were then blocked and immunoblotted with primary antibodies followed by secondary HRP-conjugated antibodies.

### 2.6. TNF-α Detection by Enzyme-Linked Immunosorbent Assay (ELISA)

HCMEC/D3 cells were stimulated, and the culture supernatants were collected as described in Section 2.5. The cytokine concentrations in the supernatants were determined by ELISA according to the manufacturer’s protocol (R&D Systems).

### 2.7. Dextran Transwell Permeability Assay

HCMEC/D3 cells were grown on 0.4 μm transwell inserts (Merck Millipore Corporation, Billerica, MA, USA) as per the manufacturer’s instructions. After the cells grew to confluence, they were exposed to 125 ng/mL nSLY or corresponding bacterial strain culture supernatants for 4 h, and at the same time, the FITC-labeled dextran (10 kDa, 100 μg/mL, Sigma, Tokyo, Japan) was added to the upper compartment and incubated together. The amount of FITC-dextran that crossed the filter to the basolateral compartment was measured with a SpectraMax^®^ i3 (Molecular Devices, San Jose, CA, USA).

### 2.8. Immunofluorescence

HCMEC/D3 cells were grown on coverslips and then stimulated by nSLY (125 ng/mL) proteins with or without 20 μM PLA2G3 inhibitor Oleyloxyethyl Phosphorylcholine (OP) for 4 h separately. Cells were then fixed with 4% paraformaldehyde and then permeabilized with 0.1% Triton X-100 for 5 min, washed in PBS for 3 times, and stained by F-actin Labeling Kit (Green Fluorescence, BBI Life Sciences Corporation, Hong Kong, China) to visualize filamentous actin (F-actin). Coverslips were mounted using DAPI (Invitrogen, Waltham, MA, USA) and analyzed using a confocal laser scanning microscope (FV1000, Olympus, Tokyo, Japan).

### 2.9. Mouse Model of S. suis 2 Meningitis

In order to further clarify the relation between suilysin and *S. suis* 2 meningitis, we inoculated Specific Pathogen Free (SPF) 7 to 8-week-old female C57BL/6 mice with 5 × 10^6^ CFU of 05ZYH33 or △SLY strains by intraperitoneal (i.p.) injection. At the same time, for the PLA2G3 inhibitor experiment, mice received daily (i.p.) injections of OP (3 mg/kg) or vehicle control for 3 consecutive days.

Bacterial loads in the blood were measured at 1, 2, and 3 days post-infection (dpi), then experimental mice were euthanized and brain tissues were harvested aseptically. Half of the brain was used to measure bacterial loads and another half was fixed in 10% buffered formalin to perform histopathological analysis. Bacterial counts in blood and brain were determined by plating serial dilutions on THB agar plates. After embedding in paraffin, tissue sections were cut and stained with hematoxylin and eosin (H&E).

### 2.10. Determination of Blood-Brain Barrier Leakage In Vivo

SPF 7 to 8-week-old female C57BL/6 mice received i.p. injections with 5 × 10^6^ CFU of 05ZYH33 or △SLY bacterial strains. At 3 days post-infection, mice were injected (i.p.) with 300 μL of 3% Evans blue (EB), which can bind albumin in serum and acts as a tracer to visualize the permeability of the BBB. The mice were sacrificed 4 h post-injection, and the brains were harvested and fixed in 10% formalin. Frozen mice brain slabs (9 μm) were kept at cryogenic temperatures until they were visualized. “Image J” was used as the integrated density analysis tool to measure extravascular accumulations of EB.

### 2.11. Statistical Analysis

GraphPad Prism 5 software was used in the data analysis by unpaired *t*-tests methods, and Kruskal–Wallis tests were used to compare the bacterial loads in blood and brain samples. Each experiment was repeated ≥3 times. For all tests, a *p* value < 0.05 was considered the threshold for significance.

### 2.12. Chemicals

SPLA2 inhibitor OP was purchased from Cayman Chemical; TNF-α inhibitor Pomalidomide was purchased from Selleckchem; PLA2G3-antibody was purchased from Sigma; cleaved caspase-3 (Asp175) antibody was purchased from Cell Signaling Technology. OP was dissolved in PBS, and Pomalidomide was dissolved in DMSO and further diluted in PBS. For vehicle controls, DMSO was diluted in PBS to the same concentration.

## 3. Results

### 3.1. SLY Induces hCMEC Overexpression of PLA2G3

**Figure 1:**
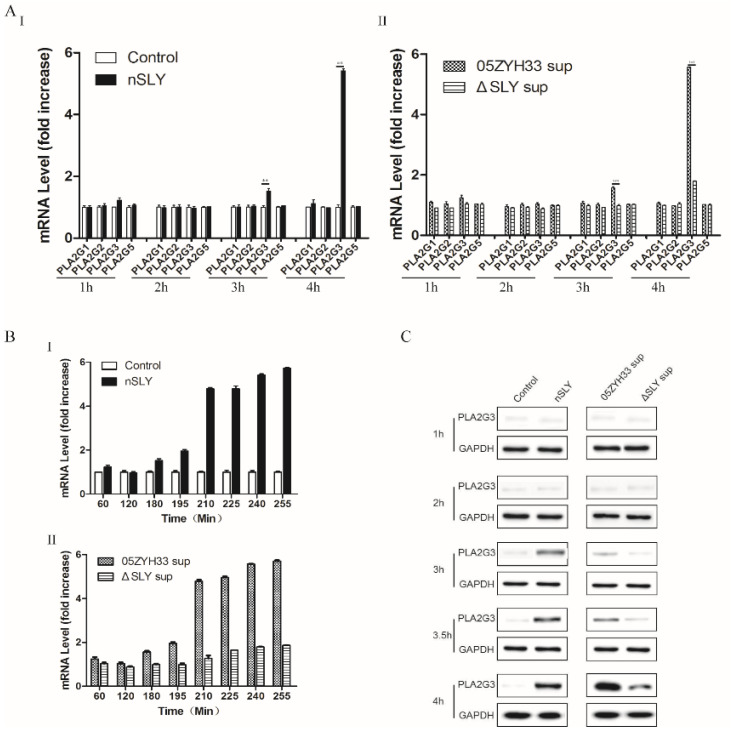
Assessing mRNA and protein expression of PLA2G3. (**A**), sPLA2 mRNA levels of Group I, II, III, and V PLA2s from 1 h to 4 h. HCMEC/D3 cells were incubated with nSLY (125 ng/mL) (**I**) or culture supernatant (**II**). (**B**) PLA2G3 mRNA expression was induced by nSLY (**I**) and culture supernatant (**II**). mRNA levels increased after 180 min incubation and exceptionally after 210 min. (**C**), PLA2G3 protein expression was assessed by Western blot analysis using anti-human PLA2G3 IgG primary antibodies to probe the blots. PLA2G3 was detected in nSLY and 05ZYH33 subgroups and the results were consistent with mRNA accumulation data. Data shown are averages of 3 independent experiments, and reported errors are SEM. Unpaired Student’s *t*-tests were used for statistical analysis. ** *p* < 0.01, *** *p* < 0.001. SEM, standard error of the mean.

Previous studies have found that pneumolysin, a member of the cholesterol-dependent cytolysin (CDC) protein family, produced by Streptococcus pneumoniae is a rapid and potent activator of cellular phospholipase A in pulmonary artery endothelial cells [22]. The SLY protein also belongs to the CDC family and was also reported to induce the release of arachidonic acid from the membrane of human brain microvascular endothelial cells [23], but the molecular mechanism responsible for this remains unknown. In this study, we evaluated if SLY could trigger the production of sPLA2 in hCMEC and the molecular mechanism responsible for this effect. To identify the sPLA2 induced by SLY in hCMEC/D3 cells, Groups I, II, III, and V sPLA2s were tested by RT-qPCR. Interestingly, only PLA2G3 exhibited an increase compared to the others after 3 h incubation (Figure 1A). PLA2G3 mRNA levels were further analyzed at subsequent time points (Figure 1B) and showed no evident difference in the first 3 h but experienced a marked increase and peak at 4 h. Western blot analysis (Figure 1C) showed that the production of PLA2G3 was consistent with its mRNA level. Collectively, these results confirm that not only nSLY but also the 05ZYH33 culture supernatant could induce an overexpression of PLA2G3 in hCMEC/D3 cells.

### 3.2. PLA2G3 Activation by SLY Occurs in a TNF-α Dependent Pathway

**Figure 2:**
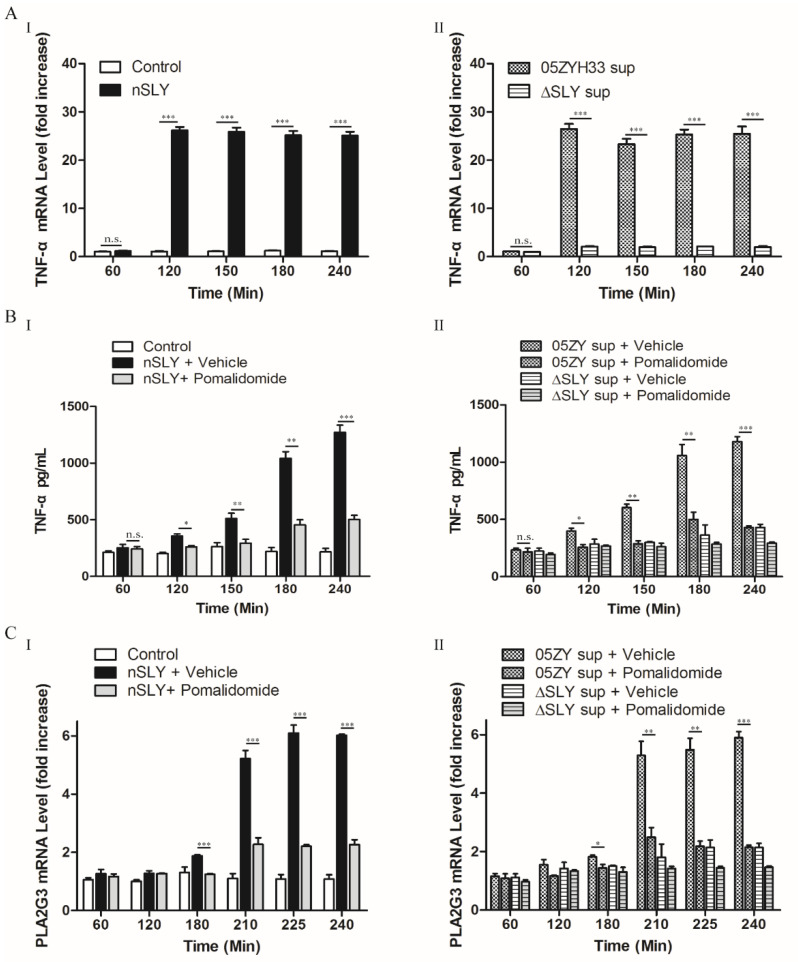
SLY-activated hCMEC/D3 cells produce PLA2G3 through TNF-α activation. (**A**) The gene expression of TNF-α in hCMEC/D3 cells induced by nSLY (125 ng/mL) (**I**) and culture supernatants (**II**). (**B**) The release of TNF-α by hCMEC/D3 was detected after stimulation with SLY, but inhibited by pomalidomide (5 μM). (**C**) PLA2G3 expression was also inhibited by pomalidomide. After the addition of the TNF-α inhibitor pomalidomide, the effect of Nsly (**I**) or 05ZYH33 sup (**II**) on PLA2G3 expression was significantly attenuated. Data are expressed as the mean ± SEM of the fold increase in mRNA above the PBS group for at least three independent experiments. * *p* < 0.05; ** *p* < 0.01, *** *p* < 0.001; n.s., no significance.

Since PLA2G3 expression did not increase until 3 h post infection (hpi), we speculated that SLY did not affect PLA2G3 expression directly and aimed to further investigate what happens within this 3 h window. As previously discussed, TNF-α induces sPLA2 expression in various cells [14,15,16]. Therefore, we determined the ability of SLY to induce TNF-α release in hCMEC/D3 cells. As shown in Figure 2A,B, SLY stimulated abundant TNF-α release for several hours, and it could be inhibited by the TNF-α inhibitor pomalidomide. Thus, we used pomalidomide to block TNF-α release to validate the role of TNF-α release in the crosstalk between SLY and PLA2G3. The results show that after TNF-α release, PLA2G3 mRNA accumulation began to increase, and its expression was efficiently downregulated with the inhibitor (Figure 2C). Therefore, in vitro assays with hCMEC/D3 cells showed that PLA2G3 activation following SLY stimulation is TNF-α dependent.

### 3.3. SLY-Mediated PLA2G3 Production Disrupted the hCMEC Monolayer Barrier

**Figure 3:**
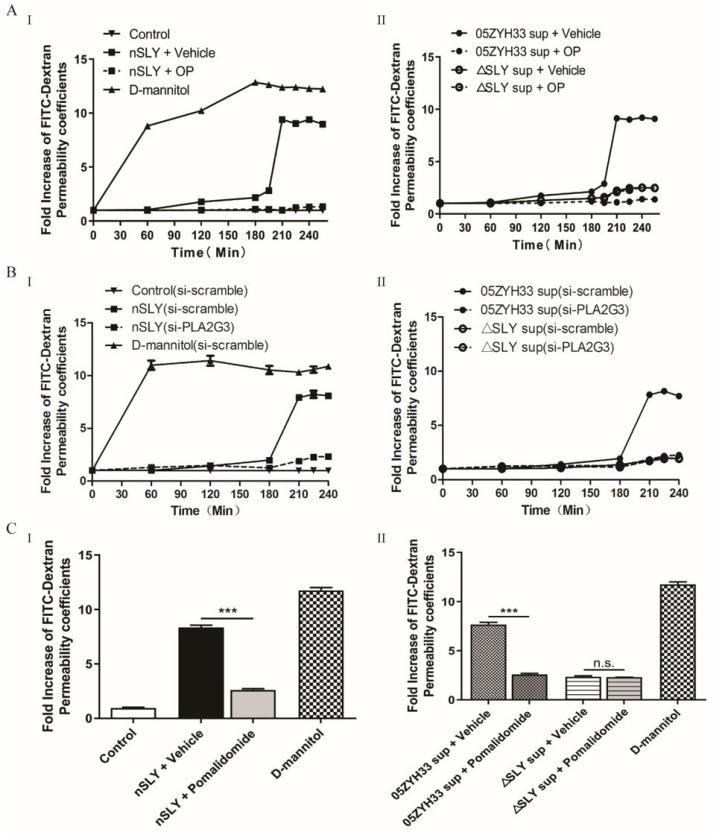
PLA2G3 increases the hCMEC/D3 cell monolayers permeability to Dextran. HCMEC/D3 cells were cultured on polycarbonate filters and stimulated with nSLY or culture supernatant in the presence of high–molecular fluorescent FITC-Dextran (100 μg/mL). The real-time measurement of FITC-Dextran in the basolateral chamber was quantified with a SpectraMax i3. Secretory phospholipase A2 inhibitor OP (20 μM) (**A**) or RNAi silencing of PLA2G3 (**B**) rescued the ability of nSLY (125 ng/mL) (**I**) and culture supernatants (**II**) to induce the high permeability of cell monolayers to Dextran. (**C**) Transendothelial permeability assay at 240 min with or without the additions of Pomalidomide (5 μM), a known TNF-α inhibitor. D-mannitol (10 μM) was used as positive control as it disrupts cell–cell junctions [26]. Data shown are averages of 3 independent experiments, and reported errors are SEM. Unpaired Student’s *t*-tests were used for statistical analysis. *** *p* < 0.001; n.s., no significance.

PLA2G3 activation might be necessary for disruption of the BBB and changing the permeability of endothelial monolayers. To test this hypothesis, the high-molecular weight fluorescent FITC-Dextran was used as a marker of tight junction integrity. Leakage of FITC-Dextran (Figure 3A) showed that SLY could significantly increase the permeability of FITC-Dextran across the hCMEC/D3 monolayer after 3 h incubation. Furthermore, we silenced PLA2G3 expression using RNA interference (RNAi) by transfecting the hCMEC/D3 cell line with siRNAs complementary to PLAG3 mRNA, which resulted in an 80% knockdown of PLA2G3 expression (Supplemental Figure S2). As expected, the use of a secretory phospholipase A2 inhibitor (Oleyloxyethyl Phosphorylcholine, OP) or silencing PLA2G3 expression rescued the SLY-induced disruption of hCMEC/D3 (Figure 3A,B). To identify whether TNF-α activity was essential for SLY-induced cytotoxicity, TNF-α inhibitor pomalidomide was added in the permeability assay (Figure 3C), and the results showed that in the presence of pomalidomide, SLY had no effect on the permeability of the endothelial monolayer. Combined, these results indicate that PLA2G3 could increase the paracellular permeability of hCMEC/D3 cell monolayers, and this interaction was dependent on TNF-α activation by SLY.

### 3.4. Increased BBB Permeability Is Dependent on SLY Triggering of PLA2G3 in S. suis 2-Infected Mice

**Figure 4:**
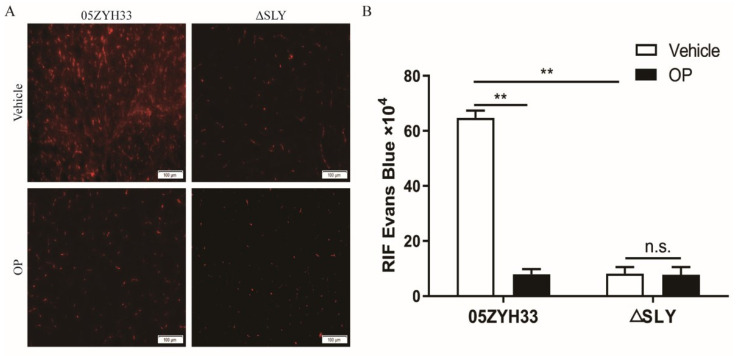
Increased BBB permeability is dependent on SLY triggered PLA2G3 expression in S. suis 2-infected mice. (**A**) Evans blue extravasation in the brains of mice 3 days post infection (dpi) with or without the addition of OP (3 mg/kg). Mean relative integrated fluorescence densities (RIF) of EB in the brain parenchyma (**B**). The brain sections depicted are from at least 4 animals in each treatment group. Data are expressed as the mean ± SEM. Unpaired Student’s t tests were used for statistical analysis, ** *p* < 0.01; n.s., no significance.

To confirm that SLY could change the vascular permeability of the brain, we used Evans blue (EB) as a tracer for in vivo experiments [27]. Three days after mice were infected with S. suis 05ZYH33 or the mutant strains, the EB solution was administered by intraperitoneal (i.p.) injection, and the mice were euthanized and the brains were removed4 h post-injection. EB was found extravasated from the microvessel in the brain parenchyma of 05ZYH33-infected mice but not in the ΔSLY-infected or secretory phospholipase A2 inhibitor OP-injected groups (Figure 4). These data show that the BBB permeability of 05ZYH33-infected mice increased, which was accompanied with PLA2G3 overexpression triggered by SLY.

### 3.5. SLY Protein Deficiency Impedes S. suis Entry into Mouse Brains

**Figure 5:**
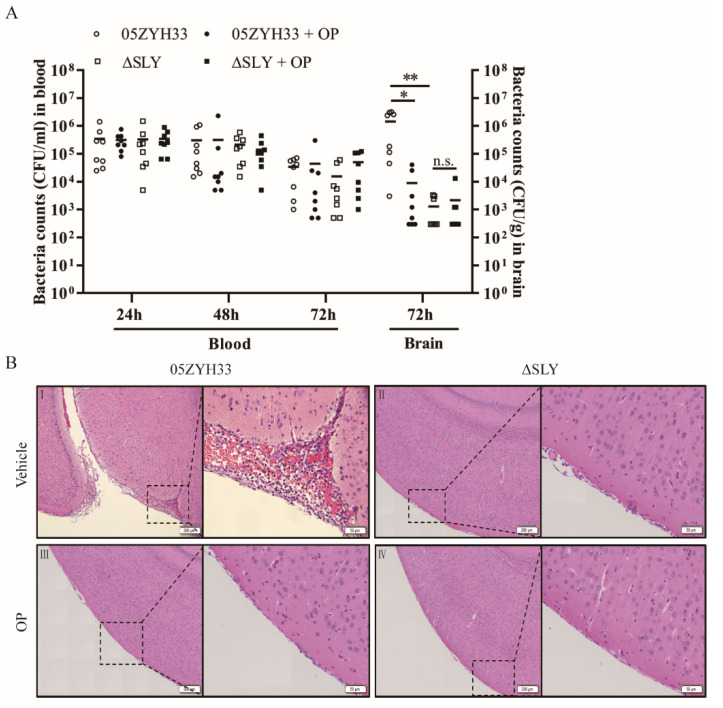
The contribution of SLY-PLA2G3 to the pathogenesis of S. suis 2 meningitis. (**A**) Bacterial concentrations in the blood and brains of mice infected with S. suis strains 05ZYH33 or ΔSLY. Each symbol represents the bacteria recovered from 1 mouse, and the horizontal lines denote the mean for each group. Kruskal–Wallis tests were used for statistical analysis. * *p* < 0.05, ** *p* < 0.01; n.s., no significance. (**B**) Histopathology of representative brain tissues from mice infected with 05ZYH33 or ΔSLY at 72 h post-infection. The tissue sections were stained with hematoxylin-eosin. Hemorrhage and meningeal thickening were observed in the WT-infected mouse (**I**) and the symptoms were attenuated by co-injection with OP (**II**). Normal brain architecture was observed in the ΔSLY mutant infected groups (**III**,**IV**). Images are representative of similar data from brains of each group.

Next, we sought to determine the effects of SLY-PLA2G3 on the ability of S. suis 2 to cause meningitis in a mouse model. In this experiment, C57BL/6 mice were divided into four groups and injected intraperitoneally with wild type S. suis 05ZYH33 (4.18 × 10^6^ CFU) or ΔSLY (4.83 × 10^6^ CFU) co-injected with OP or PBS, respectively. The bacteremia levels of the four infected mice groups were not significantly different from 1 to 3 dpi (Figure 5A). At 72 hpi, mouse brains were harvested and homogenized, and bacterial loads were counted. As shown in Figure 5A, the mean bacterial colony counts isolated from the brains of mice infected with 05ZYH33 were obviously higher compared with the ΔSLY mutant infected mice, and in agreement with the preceding results, the brain bacterial counts of animals injected with OP was lower than 05ZYH33. Moreover, histopathological examination showed that brain lesions in mice infected with 05ZYH33 displayed meningeal thickening, hemorrhaging, and severe neutrophil infiltration (Figure 5B). These animal experiments support in vitro findings that SLY can promote the development of S. suis meningitis by inducing PLA2G3 expression.

### 3.6. SLY-Mediated PLA2G3 Production Results in Apoptosis and Gap Formation

The above results demonstrated that S. suis-induced meningitis is dependent on PLA2G3 expression triggered by SLY. Next, we studied the mechanism by which PLA2G3 increases hCMEC permeability.

**Figure 6:**
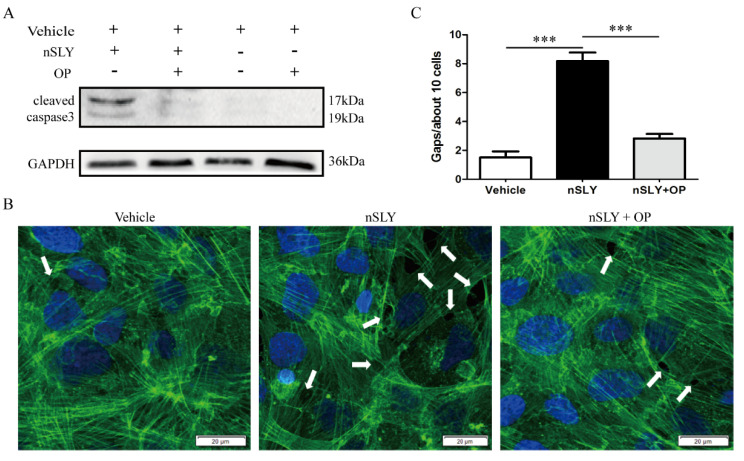
SLY-PLA2G3 induced hCMEC/D3 apoptosis and gap formation in vitro. (**A**) Caspase-3 activation by nSLY. HCMEC/D3 cells were treated with nSLY (125 ng/mL) for 4 h with or without OP. The cells were harvested and whole cell lysates were subjected to Western blot analysis using primary antibodies against the cleaved caspase-3 protein. The data shown are representative of four independent experiments. (**B**) Effects of SLY-PLA2G3 on endothelial cell intercellular gap formation. After 4 h incubation, cells were fixed, permeabilized, and stained for F-actin (green). SLY significantly induced intercellular gap formation, as indicated by the white arrows, and OP attenuated this effect. (**C**) Quantification of gaps is shown. Data are expressed as the mean ± SEM. Unpaired Student’s *t*-tests were used for statistical analysis, *** *p* < 0.001.

First, we examined the extent of apoptosis in hCMEC/D3 cells after nSLY activation in vitro. Because caspase activation is closely related to eukaryotic cell apoptosis, we analyzed whether SLY treatment could activate caspase-3, the major executioner of apoptosis. Western blot analysis showed that after nSLY treatment the amount of the cleaved, activated form of caspase-3 was obviously increased (Figure 6A). Additionally, SLY-induced apoptosis was blocked by the sPLA2 inhibitor OP. These results indicate that SLY-PLA2G3 could induce hCMEC/D3 apoptosis.

The functional integrity of the BBB is mainly regulated by the tight structure of endothelial cells. Because actin rearrangement is a primary mechanism for barrier regulation, we used immunofluorescence to examine the effects of SLY stimulation on the structure of F-actin in cultured hCMEC/D3 cells. After 4 h incubation, nSLY caused F-actin rearrangement and intercellular gap formation of hCMEC/D3 cell monolayers (Figure 6B,C). Moreover, the co-incubation of nSLY with OP blocked these effects. These structural changes have been demonstrated to cause increased permeability in cultured endothelial cells [28], which is also consistent with our prior observations.

Taken together, these results strongly suggest that SLY-PLA2G3 can induce HCMEC/D3 apoptosis and gap formation, which is associated with modifications of BBB permeability.

## 4. Discussion

Bacterial meningitis is one of the most important infectious diseases worldwide and is associated with high mortality and morbidity. Although the study of bacterial meningitis has garnered increased attention, the mechanisms by which hematogenous bacteria travel across the blood–brain barrier have not been completely elucidated [7,8]. Furthermore, *S. suis*, a previously neglected zoonotic pathogen, has recently been found to cause meningitis and associated complications in humans, especially in Southeast Asia [29].

The BBB is composed of a non-fenestrated monolayer of hCMEC, which protects the brain against microorganisms and toxins circulating in the blood. Thus, to overcome the BBB many bacterial relevant virulence factors might be involved, *S. suis* interactions with hCMEC have also been reported [23,25,30], especially those involving the SLY effector protein, which has been found to contribute to the development of *S. suis* 2 meningitis [12]. SLY is a member of the pore-forming cholesterol-dependent cytolysin (CDC) protein family, but its cytotoxicity is concentration dependent, and in vivo environments after *S. suis* 2 infection suilysin may not be able to achieve such concentrations, and as such, the subcytolytic effects of suilysin have been investigated in epithelial cell interactions of *S. suis* 2 [31]. The objective of this investigation was to determine the specific role of subcytolytic SLY in mediating hCMEC barrier disruption.

Phospholipase A2 is a protein superfamily that includes over 30 members, with sPLA2 enzymes representing the largest family [32]. In mammals, sPLA2 comprises 10 different catalytically active members (IB, IIA, IIC, IID, IIE, IIF, III, V, X, and XIIA) that vary in their tissue distribution and hydrolytic activity [33]. All sPLA2s contain a His-Asp catalytic dyad and a conserved Ca^2+^-binding loop [18]. These proteins are all of low molecular-weight (∼16 kDa) except for PLA2G3, which has a molecular weight of ∼55 kDa [34]. Interestingly, human PLA2G3 is an atypical sPLA2 that is homologous with bee venom PLA2, and its biological role remains to be analyzed [35].

The phospholipase A2 (PLA2) family can hydrolyze the *sn*-2 position of glycerophospholipids to release free fatty acids and lysophospholipids. Because of their lipase activity, sPLA2 can direct the hydrolysis of the cell membrane to increase human pulmonary endothelial permeability [36]. Moreover, the products of these enzymes are also important because they can trigger a variety of cell-signaling pathways to regulate cellular functions. sPLA2 IB and IIA proteins have been found to induce neuronal cell apoptosis, which might be associated with arachidonic acid (AA) metabolite production [37,38]. Lysophosphatidylcholine (LPC) is also a product of phosphatidylcholine hydrolysis by sPLA2, and it can impair endothelial barrier function [39]. In addition, several types of sPLA2-binding proteins have been identified, which makes it likely that these enzymes also act as ligands to receptors independent of their lipolytic enzymatic activity [40].

Some findings show that a key role of PLA2G3 is that it is involved in the initiation and/or progression of Alzheimer’s disease, which provokes apoptotic cell death [41,42]. PLA2G3 from bee venom has also been confirmed to cause rat primary cortical neuronal apoptosis [43]. Similarly to this study, our assays show that SLY-PLA2G3 could induce hCMEC apoptosis. Moreover, we also found SLY-PLA2G3 could rearrange the actin cytoskeleton of hCMEC, which lead to intercellular gap formation. All these changes induced by SLY-PLA2G3 may result in increased BBB permeability, which provides opportunities for *S. suis* 2 transmigration into the brain.

**Figure 7:**
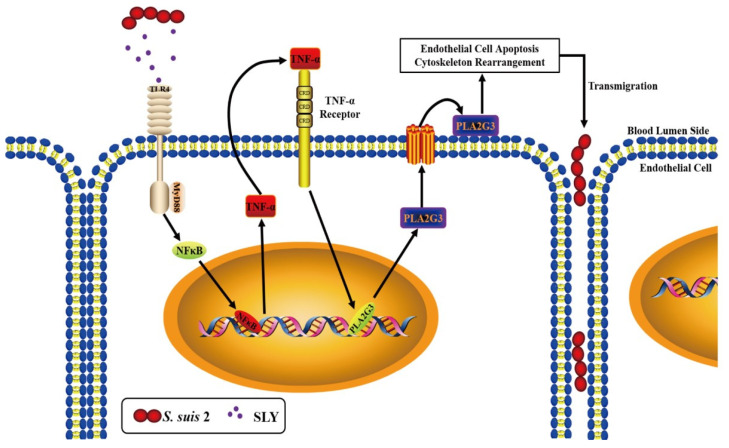
*S. suis* 2 causes meningitis through SLY-induced PLA2G3 expression. *S. suis* 2 secrete SLY in the host blood stream and subcytolytic concentrations of SLY in the blood lumen combined with receptors on endothelial cells and upregulated the expression of TNF-α and can induce the abundant release of PLA2G3 which then, through a series of coordinated actions, act to destroy the integrity of the BBB, allowing *S. suis* 2 transmigrate into the brain, leading to the development of meningitis.

To conclude, based on our previous discovery that SLY stimulated hCMEC cells to release TNF-α in a toll-like receptor 4 (TLR4)-dependent manner [13] and combined with the in vitro and in vivo results in this report, a model of *S. suis* 2 with increased endothelial permeability was proposed (Figure 7). Subcytolytic concentrations of SLY upregulated the hCMEC release of TNF-α, which led to an increased expression of PLA2G3, destroyed the integrity of the BBB, and may play an important role in the development of *S. suis* induced meningitis. Moreover, the studies described here demonstrate the linkage of SLY and PLA2G3 in these interactions.

## Data Availability

The datasets are available upon request to the corresponding author.

## Supplementary Materials

The following supporting information can be downloaded at: https://www.mdpi.com/article/10.3390/life12060919/s1, Figure S1: Determination of sub-haemolytic concentration of nSLY protein, Figure S2: Efficient attenuation of PLA2G3 expression in si-PLA2G3-transduced hCMEC/D3 cells. Table S1: Primers Used in Real-time PCR analysis and siRNAs.
