# Supplementary material for: Suilyin Disrupts the Blood–Brain Barrier by Activating Group III Secretory Phospholipase A2
Source: Life (Basel). 2022 Jun 20;12(6):919. doi: 10.3390/life12060919 (PMC9229629; doi:10.3390/life12060919)
Supplement: Supplementary file 1 [file life-12-00919-s001.zip › life-1725158-supplementary.pdf]

Supplemental Table S1. Primers Used in Real-time PCR analysis and siRNAs.

Gene	forward primer (5′- 3′)	reverse primer (5′- 3′)
TNF-α	TATGAGCCCATCTATCTG	AATGATCCCAAAGTAGAC
PLA2G1	CTATTCATACTCGTGCTCTGG	GGTCTTTCAACAAGGTGCT
PLA2G2	CTCCCTACCTAACCAAGT	ACGGAGTTGAGGTGGAGG
PLA2G3	GGACCACATCTCCTGCTGTT	CCCTAACTTCCCACCTGCTAC
PLA2G5	GATTATCATCACCACCACC	TACCCATACACCAGATTTAGAG
GAPDH	GTGGGGCGCCCCAGGCACCA	CGGTTGGCCTTGGGGTTCAGGGGGG
Si-PLA2G3	GACACCAGCUCCAGGAUAATT	UUAUCCUGGAGCUGGUGUCTT

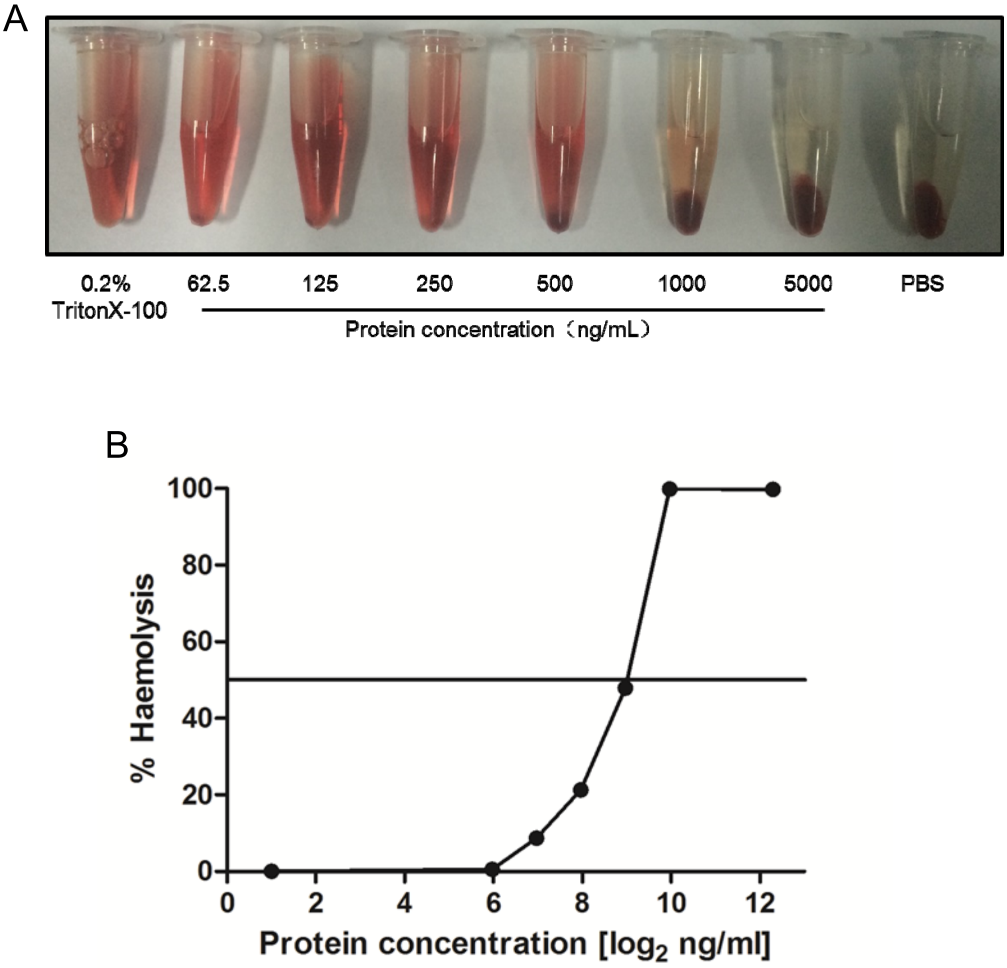

Supplemental Figure S1. Determination of sub-haemolytic concentration of nSLY protein. ***A,*** nSLY was added to 2% erythrocytes in eppendorf tube as indicated concentration and incubated at 37℃ for 60 minutes. And then the haemolysis was determined after centrifugation. ***B,*** Measuring the absorbance of supernatant at 540 nm. Results are showed as % haemolysis compared to erythrocytes lysed with 0.2% TritonX-100. The horizontal line indicates 50% haemolysis.

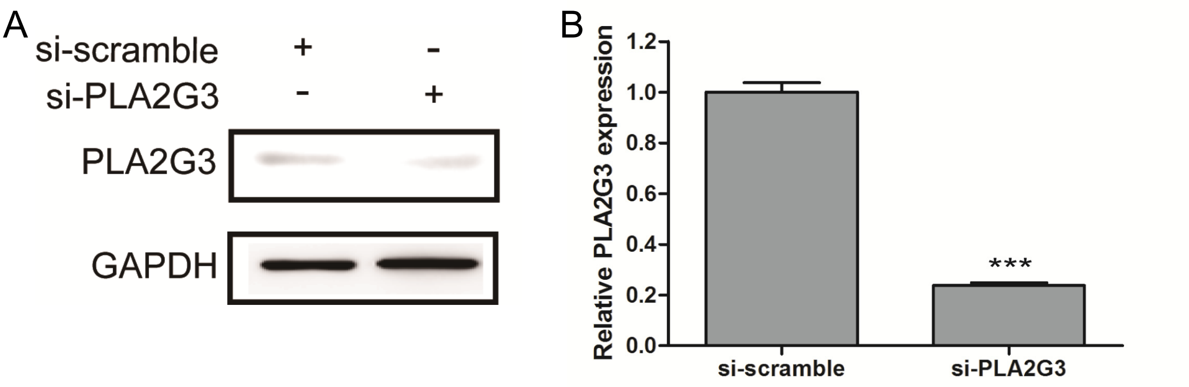

Supplemental Figure S2. Efficient attenuation of PLA2G3 expression in si-PLA2G3-transduced hCMEC/D3 cells. ***A,*** Western blot analysis of PLA2G3 in hCMEC/D3 cells after transfection with PLA2G3-targeting siRNA (si-PLA2G3) or non-targeting siRNA (si-scramble). ***B.*** QRT-PCR analysis of hCMEC/D3 cells transfection with si-PLA2G3 or si-scramble after nSLY stimulated 4 hours. Error bars, SEM; Unpaired Student t tests are used for statistical analysis. ***P <0.001.
